# Evaluating children with vestibular migraine through vestibular test battery: A cross-sectional investigation

**DOI:** 10.3389/fneur.2022.997217

**Published:** 2022-10-31

**Authors:** Fan Zhang, Jiali Shen, Qi Zhu, Lu Wang, Xiaobao Ma, Baihui He, Yang Yang, Wei Wang, Xiangping Chen, Qing Zhang, Yulian Jin, Maoli Duan, Jianyong Chen, Jun Yang

**Affiliations:** ^1^Department of Otorhinolaryngology-Head and Neck Surgery, School of Medicine, Xinhua Hospital, Shanghai Jiaotong University, Shanghai, China; ^2^Department of Otorhinolaryngology-Head and Neck Surgery, Yuyao People's Hospital, Yuyao, China; ^3^Division of Ear, Nose and Throat Diseases, Department of Clinical Science, Intervention and Technology, Karolinska Institutet, Stockholm, Sweden

**Keywords:** vestibular migraine of childhood, vestibular end organs, vestibular test battery, caloric test, video head impulse test, vestibular evoked myogenic potential, pathogenesis

## Abstract

**Objective:**

The present study aimed to investigate the status of vestibular function in children with vestibular migraine of childhood (VMC) reflected by vestibular function test battery and explore the pathophysiological implication of these instrument-based findings.

**Methods:**

The clinical data of 22 children (mean age 10.7 ± 2.9 years) with VMC who met the diagnostic criteria of the Barany Society were collected from September 2021 to March 2022. A vestibular function test battery on these children included a caloric test, video head impulse test (vHIT), cervical vestibular-evoked myogenic potential (cVEMP), and ocular vestibular-evoked myogenic potential (oVEMP); these parameters were triggered by air-conducted sound (ACS) and galvanic vestibular stimulation (GVS). The subjects were further divided into two groups: <3 months and >3 months according to the disease duration from symptom onset. The functional abnormalities and their characteristics reflected by the vestibular test battery, as well as the outcomes in children with or without aura, were analyzed.

**Results:**

(1) The abnormal rate of the caloric test was 15.8% and that of vHIT was 0%. The response rates of ACS-cVEMP and ACS-oVEMP were 100% and 90.5%, respectively. The response rates of GVS-cVEMP and GVS-oVEMP were 100% and 88.9%, respectively. (2) No statistical difference was observed in the abnormal rate of the caloric test (*P* = 0.55) and the response rate of ACS-oVEMP (*P* = 0.21) between the two groups, irrespective of the course duration. (3) No statistical difference was detected in the abnormal rate of the caloric test (*P* = 0.53) and the response rate of ACS-oVEMP (*P* = 1.00) in children with or without aura.

**Conclusion:**

Vestibular function status comprehensively reported by the vestibular test battery did not show an aggravation with the disease duration in children with VMC. Also, it was not affected by the existence of aura in children with VMC. The high abnormal rates of the caloric test and oVEMPs (ACS-oVEMP and GVS-oVEMP) suggested that the lateral semicircular canal (low-frequency function component), the utricle, and the superior vestibular conduction pathway might be involved in VMC.

## Introduction

The International Headache Society (IHS) and the Barany Society revised and published the diagnosis of vestibular migraine (VM) and probable vestibular migraine (pVM) in 2012 (1). Benign paroxysmal vertigo of childhood and VM are the most frequent pathologies leading to vertigo and dizziness during childhood (2). Previous studies have focused on the epidemiological causes of dizziness in children or initiation of the symptoms and the physical and laboratory examination and treatment in children with vestibular migraine. Studies showed that vestibular testing might have abnormal results in pediatric patients but with high variability (3–7).

Although VM is considered to be a central vestibular disorder (8, 9), peripheral vestibular end organs could also be involved (10). However, the functional abnormalities and the characteristics of the vestibular end organs in this etiological entity are not well-documented.

In 2021, the definition of vestibular migraine of childhood (VMC) and probable VMC (pVMC) was proposed based on the frequency of vestibular symptoms and the clinical signs of migraine. The diagnostic criteria of VMC are as follows (2):

A. At least five episodes with vestibular symptoms of moderate or severe intensity, lasting between 5 min and 72 h.
B. Current status or history of migraine with or without aura.
C. About 50% of the episodes are associated with at least one of the following three migraine features:
  1. Headache with at least two of the following four characteristics:
    a) One-sided location.
    b) Pulsating quality.
    c) Moderate or severe pain intensity.
    d) Aggravation by routine physical activity.
  2. Photophobia and phonophobia
  3. Visual aura.
D. Age < 18 years
E. Not better accounted for by another headache disorder, vestibular disorder, or other condition.

Moreover, the applications of galvanic vestibular stimulation-vestibular-evoked myogenic potentials (GVS-VEMPs) in children are being explored. This could help in locating the lesions combined with air-conducted sound (ACS)-VEMPs; however, there are no reports on the GVS-VEMP results in children with VM.

In this study, we conducted the vestibular test battery to explore the putative vestibular pathway involved in patients with VMC according to the new diagnostic criteria.

## Patients and methods

The present study was approved by the Ethics Committee of Xinhua Hospital, Shanghai Jiao Tong University, School of Medicine (No. XHYY-2021-039), and informed consent was obtained from the children's guardians. The clinical data of 22 children with dVMC who visited the Department of Otorhinolaryngology-Head and Neck Surgery of Xinhua Hospital, Shanghai Jiao Tong University, School of Medicine, were collected from September 2021 to March 2022 and analyzed retrospectively.

The inclusion criteria for VMC were as follows: (1) children who met the diagnostic criteria of the Barany Society in 2021 (2); (2) children who had completed the vestibular test battery.

The exclusion criteria were as follows: (1) external or middle ear diseases; (2) other definite vestibular diseases; (3) structural abnormalities on brain magnetic resonance imaging (MRI) and/or electroencephalogram (EEG) findings indicative of vertiginous epilepsy; (4) vertigo-related diseases of other systems, such as neurological and psychiatric.

The parents were asked to provide their medical history in detail. All patients completed pure-tone audiometry, cranial MRI, electroencephalogram, and vestibular test battery including caloric test, video head impulse test (vHIT), cervical VEMP (cVEMP), and ocular VEMP (oVEMP), triggered by ACS and GVS, respectively.

In terms of technical feasibility, the cVEMP test can be conducted in newborns, whereas oVEMP is not performed until the age of 3 years (11, 12). vHIT can be performed in infants >3 months old, while the caloric test is routinely conducted in children >6 or 7 years old due to less impact of fear and focus at that age (13, 14). Therefore, all tests in the vestibular test battery are suitable for children in the age range in this study.

### Audiometry

Pure-tone audiometry was conducted in a soundproof room using an audiometer (Type Astera, Madsen, Denmark). The pure-tone average (PTA) is the average of the 0.5, 1, 2, and 4 kHz air-conduction thresholds. PTA <20 dB HL is considered normal (15). The air-bone gap (ABG) is calculated as the air-conduction threshold minus the bone-conduction threshold at the same pure-tone frequency (16).

### Vestibular testing

#### Caloric test

The patients lay supine in a dark room and looked straight ahead with their heads elevated 30° to keep the horizontal semicircular canal vertical to the ground. Any spontaneous nystagmus was recorded by video-nystagmography (Interacoustics). Cold (24°C) and hot (50°C) air irrigations were completed in both external auditory canals, both for 60 s sequentially (13, 17). Then, the nystagmus was recorded, and the percentage of canal paresis (CP%) and dominant preponderance (DP%) was calculated using Jongkees' formula. The caloric test was defined as abnormal if CP was >25% and/or bithermal peak slow phase velocity (SPV) on each side was <6°/s or DP was >30% (5, 18).

#### Video head impulse test

A video head impulse test was performed using a video head pulse instrument (Interacoustics, EyeSeeCam, Denmark). The patients were seated in a chair, looked straight ahead at a fixed visual target 1 m in front of their eyes, avoided blinking, and relaxed their neck muscles. An experienced technician delivered at least 20 high-acceleration, sudden, and unpredictable head impulses per side (10–20°, duration 150–200 ms, peak velocity of >150°/s for horizontal head impulses, and >100°/s for vertical head impulses) (13, 19). An instantaneous vestibulo-ocular reflex (VOR) gain was automatically calculated using the equipment software, which is eye velocity (°/s)/head velocity (°/s). Abnormal vHIT was defined when the instantaneous VOR gain at 60 ms for the horizontal canal was <0.8 or the regression VOR gain for vertical canals was <0.7 or showed corrective saccades in each semicircular canal (20).

#### ACS-cVEMP

ACS-VEMP test was examined using Neuropack MEB-9404C (NIHON KOHDEN, Japan). Short pure tones (500 Hz, 105 dB nHL intensity, rise/fall time 1 ms, plateau period 2 ms, superposition 50 times, window opening time 0–60 ms, stimulation rate 5.1 times/s, impedance <10 kΩ) were presented monaurally through a calibrated headphone TDH-39. The recording electrodes were placed on the upper third of the bilateral sternocleidomastoid muscles (SCMs). The reference electrode was placed between the clavicle joints. Then, the ground electrode was placed in the middle of the forehead. The patients were in a supine position and asked to raise their heads 30° upon the horizon to keep the SCM tense from the start of a single stimulus sound until the end. The electromyographic monitoring limited the variability and guaranteed bilateral muscle tones in case the children could not cooperate during the test.

For better observation of the waveforms, each grid represented 5 ms on the horizontal axis and 100 or 200 μV on the vertical axis. A positive wave of 13 ms after stimulation was labeled as p1, and a negative wave of 23 ms was labeled as an n1 wave. cVEMP was defined if reproducible p1 and n1 waveforms could be elicited, and no response was defined as the absence of meaningful p1 and n1 waveforms. Latency of p1 and n1 waves and amplitudes of p1-n1 were obtained, defined as the vertical distance between the highest point of the p1 wave and the lowest point of the n1 wave. The asymmetry ratio (AR) was calculated using the large p1-n1 amplitude (AL) and the small p1-n1 amplitude (AS) and the following formula: AR (%) = (AL-AS)/(AL+AS) × 100%. A difference of >40% between two ears is considered significant, while no response or AR (%) of >40% was considered abnormal in this study (13, 21).

#### ACS-oVEMP

The parameter settings of ACS-oVEMPs were similar to those ACS-cVEMP, while electromyographic monitoring was not required. The recording electrode was placed 1 cm below the middle of the contralateral eyelid, the reference electrode was placed 2 cm below the recording electrode, and the ground electrode was placed in the middle of the forehead. The patients were asked to maintain eye gaze upward for 25–30° after hearing a single acoustic stimulus and minimize blinking to maintain tension in the inferior oblique muscle until the stimulation stops. As for the waveforms, each grid represented 5 ms on the horizontal axis and 5 or 10 μV on the vertical axis. A negative wave occurring about 10 ms after stimulation was labeled as an n1 wave, and a positive wave at about 16 ms was labeled as a p1 wave. Then, oVEMP was confirmed to be induced if reproducible n1 and p1 waveforms could be elicited. No response or AR (%) >40% was considered abnormal (13, 21).

#### GVS-cVEMP

GVS-cVEMP was examined using an electrophysiological recorder (Neuropack MEB-9404C, NIHON KOHDEN, Japan). The recording electrodes were placed at the upper third of the bilateral SCMs, the reference electrode was placed between the clavicle joints, and the ground electrode was at the nasal root. The cathode of the direct current stimulation was placed at the mastoid process, and the anode was placed at the midpoint of the forehead hairline. The initial stimulation (3.0 mA, stimulation rate 5 Hz, band-pass filter 20–2,000 Hz, superposition 50 times, and time window 50 ms) was direct current, and the waveform was recorded using an electromyographic amplifier. The waveform under muscle contraction was subtracted from the waveform under muscle relaxation to eliminate the artifacts of mechanical waves (22–24). If the reproducible waveform could not be elicited at 3.0 mA, the stimulation could rise according to the patient's tolerance, typically at ≤ 5.0 mA. Then, the latency of p1 and n1 waves, amplitudes of p1-n1, and AR% were recorded.

#### GVS-oVEMP

The recording electrode was placed 0.5–1.0 cm below the eyelid, the reference electrode was placed 2.0 cm below the recording electrode, and the ground electrode was placed at the nasal root. The cathode of the direct current stimulation was placed at the mastoid process and the anode at the midpoint of the forehead hairline. The waveform obtained from eye gaze upward for 25–30° was subtracted from the waveform obtained from eye gaze downward to eliminate mechanical wave artifacts (22, 23).

### Data analysis

All data were analyzed using SPSS v.22 statistical software. The categorical variables were expressed as a ratio, while the continuous variables were expressed as mean ± standard deviation. The abnormal rates and response rates of vestibular testing were compared by Fisher's exact test. *P* < 0.05 was considered statistically different.

## Results

### Characteristics

The clinical and demographical data of the 22 children included in this study are listed in Table 1. The average age of the cohort was 10.7 ± 2.9 (range: 6–17) years. A subset of the children with VMC presented migraine features in the episodes: aura occurred in 5, headache aggravated by routine physical activity or stress in 3, photophobia and/or phonophobia in 8, motion sickness in 7, and a family history of migraine in 7. All had auditory thresholds within normal range, with PTA < 20 dBHL and no ABG. EEG did not find any spikes and sharp waves or other epileptiform discharges, and two patients showed mild abnormalities. Brain MRIs were normal.

**Table 1:** Characteristics of patients with VMC.

**Characteristics**	***N* = 22**
Age (years)	10.7 ± 2.9
**Sex**
Male	14 (63.6%)
Female	8 (36.4%)
Aura	5 (22.7%)
Headache aggravated by daily physical activity or stress	3 (13.6%)
Photophobia and/or phonophobia	8 (36.4%)
Motion sickness	7 (31.8%)
Family history	7 (31.8%)

### Rates of abnormal vestibular tests

**Figure 1:**
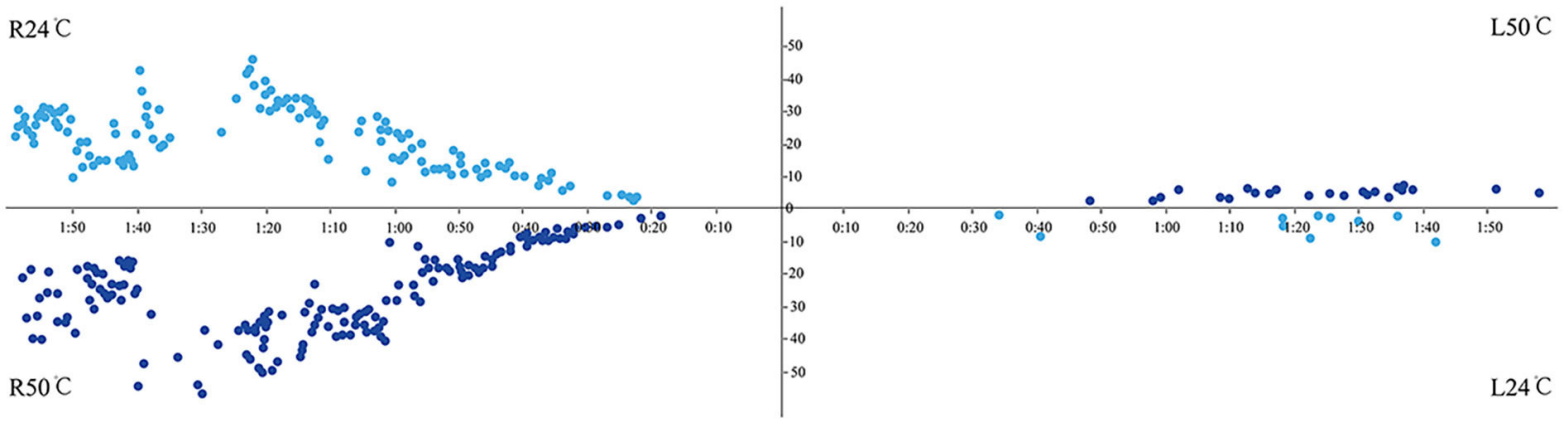
Abnormal caloric test showed attenuation on the left side.

**Figure 2:**
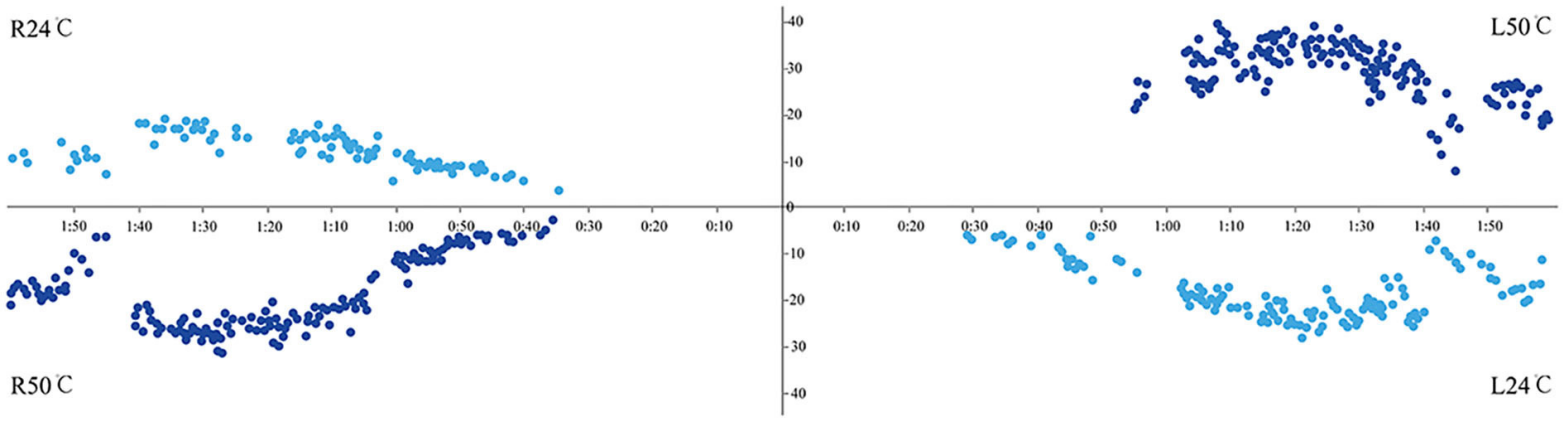
Normal caloric test showed bilateral symmetry and no absolute caloric hypofunction.

**Figure 3:**
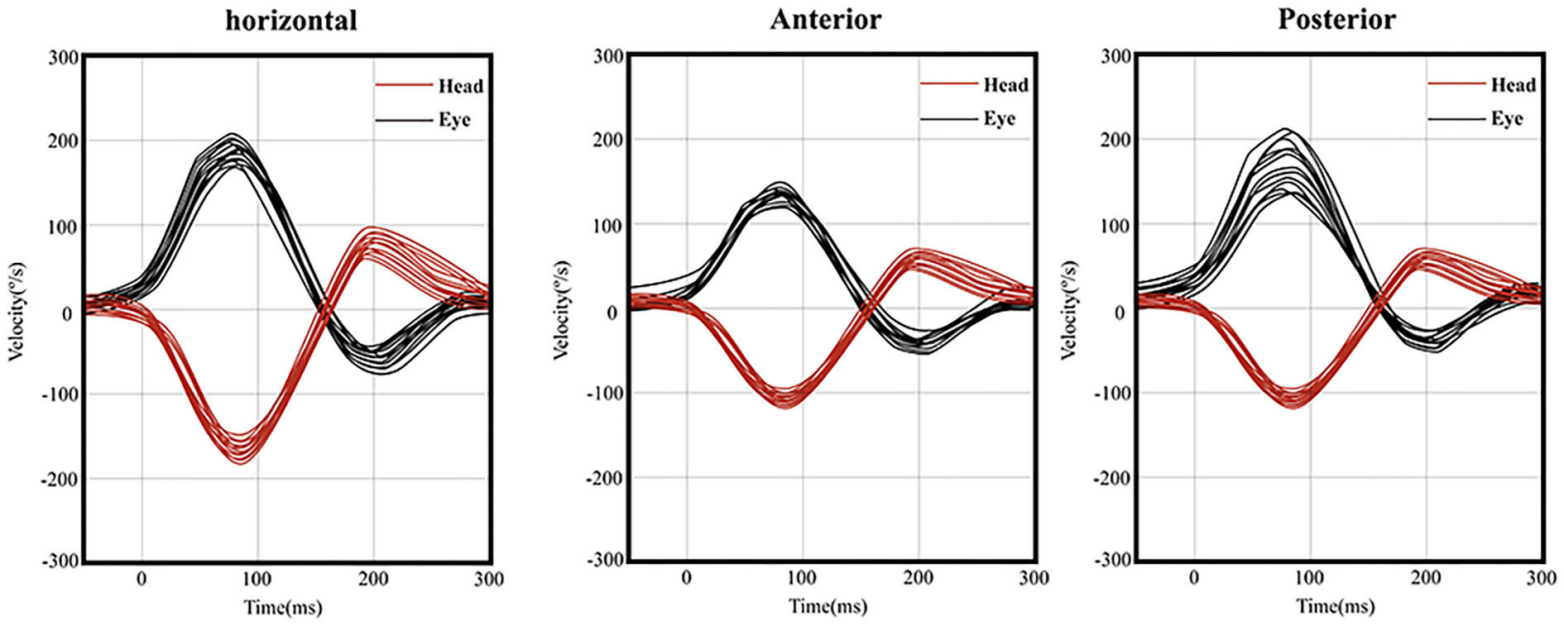
Normal vHIT on the right side of one patient. **(left)** Instantaneous VOR gain at 60 ms for the horizontal canal was 1.02 and showed no corrective saccades. **(middle)** Regression VOR gain for the anterior canal was 1.06 and showed no corrective saccades. **(right)** Regression VOR gain for the posterior canal was 1.45 and showed no corrective saccades.

Not all children completed the entire test battery but most of the tests in the protocol were completed. The results showed that the abnormal rates of the caloric test and vHIT were 15.8% (3/19) and 0% (0/22), respectively. Among them, one patient showed CP >25% (Figure 1), two showed DP >30%, and three had spontaneous nystagmus with SPV = 1, 1, and 2°/s, respectively (< 3°/s). The typical normal caloric test and vHIT testing are shown in Figures 2 and 3.

**Figure 4:**
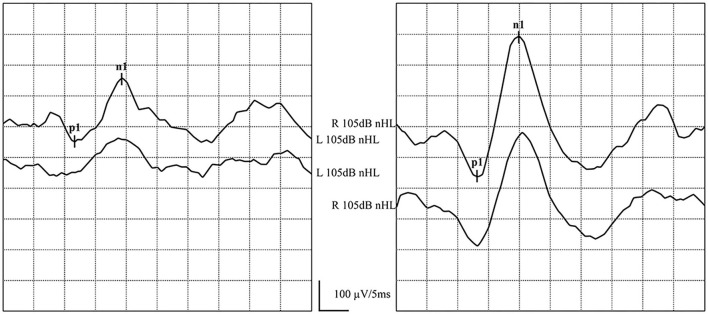
Normal ACS-cVEMP waveforms: Elicited bilaterally and AR = 38%. **(left)** ACS-cVEMP waveforms were elicited on the left side. **(right)** ACS-cVEMP waveforms were elicited on the right side.

**Figure 5:**
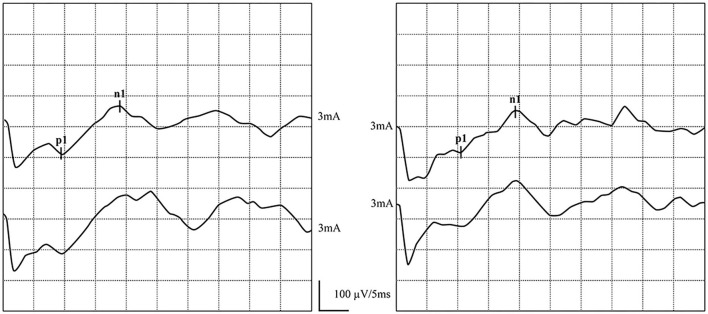
Normal GVS-cVEMP waveforms: Elicited bilaterally. **(left)** GVS-cVEMP waveforms were elicited on the left side. **(right)** GVS-cVEMP waveforms were elicited on the right side.

The response rates of ACS-cVEMP and ACS-oVEMP were 100% (20/20) and 90.5% (19/21), respectively. The response rates of GVS-cVEMP and GVS-oVEMP were 100% (18/18) and 88.9% (16/18), respectively. No statistically significant difference was observed between the two groups (*P* = 0.48, 0.49). The response rates of ACS-cVEMP, ACS-oVEMP, GVS-cVEMP, and GVS-oVEMP are listed in Tables 2 and 3. The typical normal ACS-cVEMP and GVS-cVEMP are shown in Figures 4 and 5.

**Table 2:** Response rates of ACS-cVEMP and ACS-oVEMP.

**ACS-cVEMP**		**Left**		**ACS-oVEMP**		**Left**	
		**Elicited**	**Not elicited**			**Elicited**	**Not elicited**
Right	Elicited	20	0	Right	Elicited	19	1
	Not elicited	0	0		Not elicited	0	1

**Table 3:** Response rates of GVS-cVEMP and GVS-oVEMP.

**GVS-cVEMP**		**Left**		**GVS-oVEMP**		**Left**	
		**Elicited**	**Not elicited**			**Elicited**	**Not elicited**
Right	Elicited	18	0	Right	Elicited	16	2
	Not elicited	0	0		Not elicited	0	0

**Figure 6:**
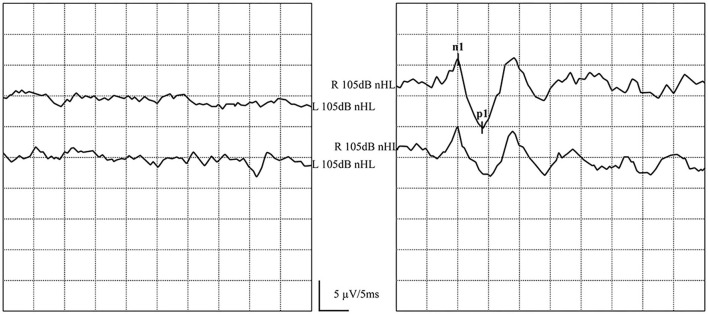
Normal and abnormal ACS-oVEMP waveforms: Not elicited on the left side and elicited on the right side. **(left)** Abnormal ACS-oVEMP waveforms: Not elicited on the left side. **(right)** Normal ACS-oVEMP waveforms: Elicited on the right side.

**Figure 7:**
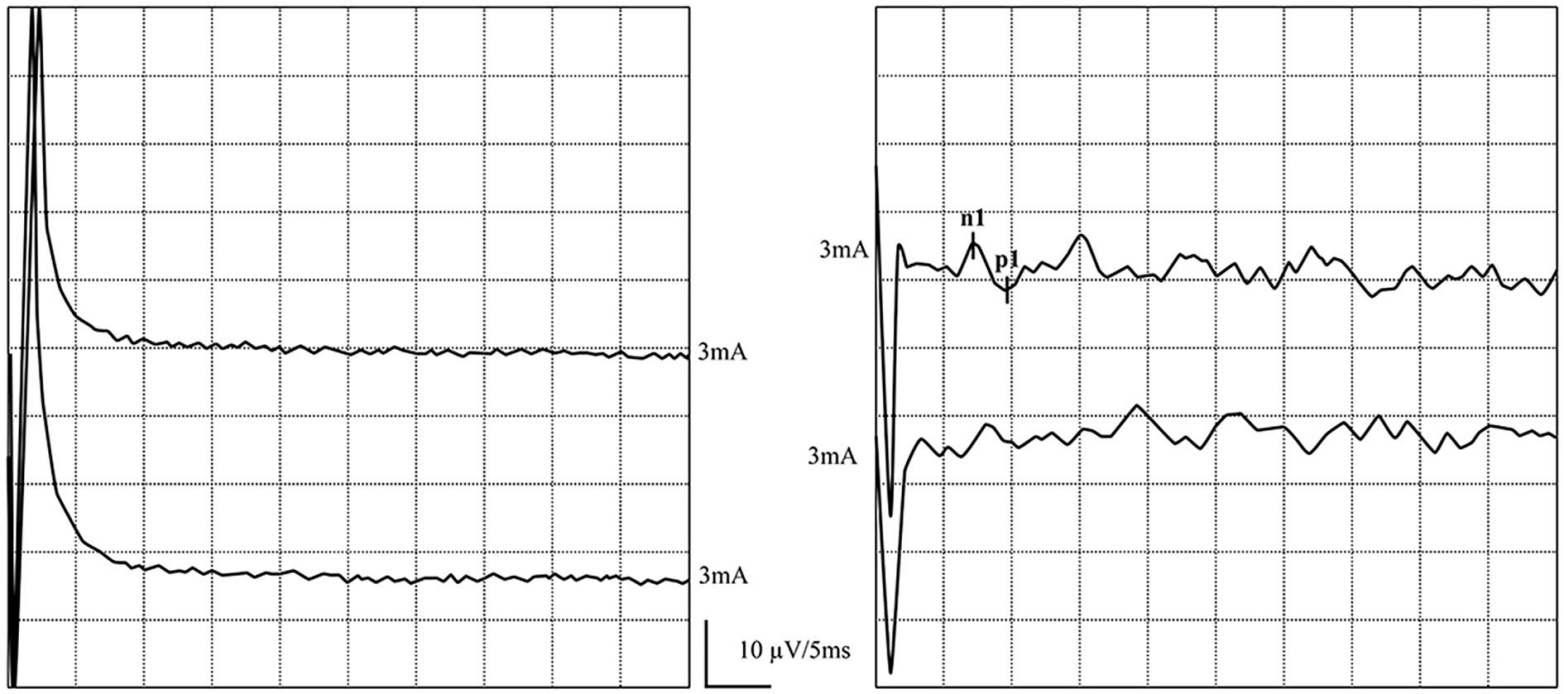
Normal and abnormal GVS-oVEMP waveforms: Not elicited on the left and elicited on the right side. **(left)** Abnormal GVS-oVEMP waveforms: Not elicited on the left side. **(right)** Normal GVS-oVEMP waveforms: Elicited on the right side.

In one patient, ACS-oVEMP was not elicited bilaterally, and GVS-oVEMP was not elicited on the left side. In another patient, ACS-oVEMP was not elicited on the left side (Figure 6), but GVS-oVEMP was elicited bilaterally. Another patient showed bilaterally elicited ACS-oVEMP, but GVS-oVEMP was not elicited on the left at 3.0 mA (Figure 7).

In addition, one child showed AR > 40% in ACS-oVEMP, and thus, the abnormal rates of ACS-cVEMP and ACS-oVEMP were 0% and 14.3% (3/21), respectively.

### Course duration, aura, and vestibular tests

The mean duration of the disease was 667 ± 1,061 days, ranging from 6 days to 10 years. The children were divided into <3 months and >3-month groups according to the disease duration from symptom onset. The former consisted of 10 patients and the mean disease duration was 27.4 ± 16.1 days, while the latter had 12 patients and the mean duration was 1,146 ± 1,209 days. The comparison of the abnormal or the response rates of vestibular test battery between the two groups, irrespective of the course duration, is shown in Table 4. No statistical difference was found in the abnormal rate of the caloric test (*P* = 0.55) and the response rate of ACS-oVEMP (*P* = 0.21) and GVS-oVEMP (*P* = 1.00) between the two groups.

**Table 4:** Comparison of the abnormal rates or the response rates of vestibular testing according to the course duration.

**Group**	**Caloric test**		**ACS-oVEMP**		**GCS-oVEMP**	
	**Normal**	**Abnormal**	**Elicited**	**Not elicited**	**Elicited**	**Not elicited**
Course duration < 3 months	6	2	8	2	8	1
Course duration >3 months	10	1	11	0	8	1
*P*	0.55		0.21		1	

The vestibular test battery results in children with aura (*n* = 5) were normal, and the comparison with children with no aura is shown in Table 5. No statistical difference was found in the abnormal rate of the caloric test (*P* = 0.53) and the response rate of ACS-oVEMP (*P* = 1.00) and GVS-oVEMP (*P* = 1.00) between the two groups.

**Table 5:** Comparison of children with or without aura.

**Group**	**Caloric test**		**ACS-oVEMP**		**GCS-oVEMP**	
	**Normal**	**Abnormal**	**Elicited**	**Not elicited**	**Elicited**	**Not elicited**
Aura	5	0	5	0	5	0
No aura	11	3	14	2	11	2
*P*	0.53		1		1	

## Discussion

The prevalence of vestibular disorders in the pediatric population is 0.7–15% (7, 25, 26). Migraine-related vertigo is the most common diagnosis in 7–12-year-old children (7). The mean age of the patients in our study was 10.7 ± 2.9 years, which was similar to the previous study. In a study of child migraine sufferers with vestibular symptoms, many children reported “the home is moving” or “the picture is moving” already at 3–4 years of age (10). We also reported vestibular symptoms at an early age in children, with a duration of 667 ± 1,061 days.

### The putative pathophysiological mechanism of VM

Currently, the pathophysiological mechanism of VM is unclear. Nonetheless, many similarities have been detected in the mechanisms underlying VM and migraine; animal experiments have demonstrated that the brain stem is involved in the pathophysiological mechanism of migraine (10). The persistent brain stem activation after injecting sumatriptan supports the explanation of an imbalance in the activity between brain stem nuclei regulating antinociception and vascular control in migraine (27). Another hypothesis is that the triggering of the trigeminal-vestibulocochlear reflex increases the blood flow in the inner ear, releases active substances, and extravasates plasma protein, which could produce neurogenic inflammation to sensitize the first/second/third-order trigeminovascular neurons causing allodynia, followed by the manifestation of the VM symptoms (28).

### VEMPs and the underlying pathway

Marcelli et al. (10) speculated that both central vestibular and peripheral pathways participate in the etiopathology in children with migraine without the involvement of the auditory pathway. Although the diagnosis of dizziness in children like VMC mainly depends on their medical history, the results of the vestibular test battery provide information about various underlying vestibular pathways. These tests could help in the differential diagnosis of dizziness in children (4, 6), although many studies presented almost normal results. For example, abnormal vestibular test results suggested a vestibular disorder rather than psychological problems, developmental disorders, torticollis, and ataxia (13).

According to the conduction path, ACS-cVEMP could be used to assess the pathway including the saccular and the inferior vestibular nerve; ACS-oVEMP could be used to assess the pathway including the utricle and the superior vestibular nerve (13). Some studies reported reduced amplitude or delayed latency of VEMP responses, while others found asymmetric ACS-VEMP responses with normal latency and amplitude (29). Similarly, O'Reilly et al. (3) and Brodsky et al. (4) reported normal cVEMP results in all 25 and 16 pediatric patients with VM, respectively, while Langhagen et al. (5) demonstrated abnormal cVEMP results in 33% of children diagnosed with VM. Notably, oVEMP testing has rarely been reported previously because it is challenging to implement in the evaluation of patients with VMC. In the present study, the response rates of ACS-cVEMP and ACS-oVEMP were 100% (20/20) and 90.5% (19/21), respectively. The normal cVEMP results indicated that the pathway from the saccule, inferior vestibular nerve, vestibular nucleus, accessory nucleus, and accessory nerve to sternocleidomastoid muscle was intact. The higher abnormal rate of oVEMPs suggested that the utricle and the superior vestibular conduction pathways might be involved and impaired in VMC.

Opposite to ACS, GVS directly stimulates the vestibular afferent nerve. Compared to ACS-cVEMP, GVS-cVEMP provides locational information about whether a lesion is located in the labyrinth or retrolabyrinth (23, 30, 31). Hitherto, no studies have reported the use of GVS-VEMP in evaluating vestibular function in children. Herein, we obtained a reproducible GVS-VEMP waveform successfully. The response rate of GVS-cVEMP was 100% (18/18), consistent with ACS-cVEMP, and that of GVS-oVEMP was 88.9% (16/18), partially different from the results of ACS-oVEMP. Based on the current results, the deficit of the vestibular nerve or the receptor organs could not be determined. Moreover, in one 17-year-old patient, ACS-oVEMP was elicited bilaterally, but GVS-oVEMP was not elicited on the left at 3.0 mA. This could be attributed to insufficient electrical stimulation. Zhang et al. (24) demonstrated that with increasing age, the response rate decreased and the threshold increased.

### Caloric test, vHIT, and the underlying pathway

The caloric test provides ear-specific, low-frequency information about the horizontal semicircular canal and the superior branch of the vestibular nerve. vHIT testing shows the function of the semicircular canals at high frequency and both branches of the vestibular nerve (13, 32). The results of the previous studies on the caloric test and vHIT in patients with VMC were interpreted as normal, hyperreflexia, or weak, and the abnormality of vHIT was always low. Conversely, O'Reilly et al. (3) found normal results on the caloric test. Duarte et al. (33) demonstrated mostly normal or bilateral hyperreflexia in the caloric test. Another study on migraine sufferers with vestibular symptoms also showed bilateral weakness in 25% of children and unilateral weakness in 19% of children in the caloric tests, wherein the high abnormality could be due to a larger study population than in studies about VMC (10). Furthermore, Langhagen et al. (5) found that the abnormal rate of the caloric test and vHIT was 21% and 8%, respectively. In the current study, we observed partially reduced caloric response but no vHIT abnormality. The abnormal rate of the caloric test and vHIT was 15.8% (3/19) and 0% (0/22), indicating that the lateral semicircular canal (low-frequency function component) may be involved in VMC. Halmagyi et al. (34) speculated that isolated DP reflects a gain asymmetry between the neurons in the medial vestibular nucleus on either side, suggesting a status of vestibular decompensation.

### Course duration, aura, and vestibular testing

While vestibular abnormalities are often found in patients with VM (35–37), high normal rates of vestibular function tests are observed in patients with VMC. This phenomenon could be explained based on the fact that abnormalities in vestibular function testing in patients with VM might result from ischemic damage due to long-term disease, whereas VMC children may have less time to develop such changes (4). Some studies used MRI-based voxel-based morphometry to evaluate patients with VM and found brain structural changes. The increased or decreased gray matter volume was related to self-adaptation of the nervous system or transmission circuitry impairment in the central vestibular cortex, respectively (38, 39). Obermann et al. (8) established a negative correlation between disease duration and gray matter volume in areas associated with headache and vestibular processing, indicating a pathophysiological change affected by the disease duration in patients with VM. However, in the two groups of patients with a duration of < or >3 months in this study, we did not find any statistical difference in the abnormal rate of vestibular testing. Also, vestibular function status did not aggravate disease duration in children with VMC.

Dizziness and vertigo are frequently associated with migraine. A study in 2010 on 22 migraine-suffering children with vestibular symptoms demonstrated that the vestibular test, including bithermal caloric test and VEMPs, was abnormal in 100% of children (10/10) with aura, indicating a significant involvement of vestibular pathways compared to 50% (6/12) positive children without aura (10). However, no difference was observed in the vestibular testing outcomes in children with or without aura. In the current study, the dVMC population was included according to the diagnostic criteria of the Barany Society in 2021, which might vary from the grouping criteria described above.

### Limitations

The main limitations of this study are as follows: (1) a control group of healthy children was not recruited, thus lacking normal values of vestibular testing in children with matched age, which affected the determination of abnormal rates. (2) The number of study subjects was small and needs to be expanded for reliable statistical results. (3) Side differences in cVEMPs might be due to different muscle tones on the two sides as no corrected amplitudes were used.

## Conclusion

Vestibular function status could be comprehensively reported by the vestibular test battery. The high abnormal rates of the caloric test and oVEMPs (ACS-oVEMP and GVS-oVEMP) suggested that the lateral semicircular canal (low-frequency function component), the utricle, and the superior vestibular conduction pathway might be involved in VMC. The vestibular function was neither aggravated with disease duration nor was affected by aura in children with VMC.

## Data availability statement

The raw data supporting the conclusions of this article will be made available by the authors, without undue reservation.

## Ethics statement

The studies involving human participants were reviewed and approved by the Ethical Committee of the Xinhua Hospital. Written informed consent to participate in this study was provided by the participants' legal guardian/next of kin. Written informed consent was obtained from minor(s)' legal guardian/next of kin, for the publication of any potentially identifiable images or data included in this article.

## Author contributions

JY, MD, and JC contributed to the study design and reviewed and approved the final manuscript. FZ and JS contributed to the detailed study design, statistical analysis, and manuscript draft and revision. LW, XM, and WW performed the vestibular function tests. BH and YY contributed to data acquisition. QZha, XC, QZhu, and YJ critically reviewed the manuscript. All authors agree to be accountable for the content of this study and the integrity and accuracy of the data. All authors contributed to the article and approved the submitted version.

## Funding

This study was supported by the National Natural Science Foundation of China (No. 81873698, 2019), the Science and Technology Commission Foundation of Shanghai (No. 21Y31900504), and the Hospital Funded Clinical Research, Xin Hua Hospital Affiliated to Shanghai Jiao Tong University School of Medicine, Clinical Research Unit (No. 21XHDB02, 2021).

## Conflict of interest

The authors declare that the research was conducted in the absence of any commercial or financial relationships that could be construed as a potential conflict of interest.

## Publisher's note

All claims expressed in this article are solely those of the authors and do not necessarily represent those of their affiliated organizations, or those of the publisher, the editors and the reviewers. Any product that may be evaluated in this article, or claim that may be made by its manufacturer, is not guaranteed or endorsed by the publisher.
